# Failure Analysis of Abnormal Cracking of the Track Circuit Reader Antenna Baffle for High-Speed Trains

**DOI:** 10.3390/ma16020722

**Published:** 2023-01-11

**Authors:** Chang Su, Tong-Tong Bi, Zhen-Guo Yang

**Affiliations:** Department of Materials Science, Fudan University, Shanghai 200433, China

**Keywords:** polyurethane rubber, antenna baffle, environmental stress cracking, bubble, failure analysis

## Abstract

The track circuit reader (TCR) is an important part of train control systems. This paper reports a failure of the TCR antenna baffle, which is used to prevent the TCR antenna from being struck by foreign objects. The designed service life of the baffle is 4.8 million kilometers, but serious cracking was found during routine maintenance after only 0.67 million kilometers of operation. In order to avoid the hidden danger brought by the incident to the safe operation of the train, it is necessary to conduct a complete failure analysis of the failed TCR antenna baffle. Therefore, a comprehensive investigation of the base material, cleaning agents, crack morphologies, etc., was carried out, and the failure environment of the antenna baffle was verified by experiment. The final results show that the environmental stress cracking is the root cause of the failed antenna baffle, and the multiple bubbles produced by the formed process of the antenna baffle are another important cause. According to the conclusions, the solutions to prevent the reoccurrence of such failures are proposed. After these solutions are adopted, the number of failed antenna baffles is greatly reduced, which fully proves the correctness of this analysis.

## 1. Introduction

The track circuit reader (TCR) is a signal subsystem used on some electric multiple unit (EMU) trains with speeds over 300 km/h. As an important part of the train control system, it reads the relevant track circuit information code and provides normal or braking information for the onboard safety computer. According to the output information of the TCR and the information of the ground transponder, the train control system ensures the security of train operation. The TCR antenna is the exposed part of the TCR device. In order to prevent the TCR antenna from being struck by foreign objects when the train is running, a baffle is installed on the side of the TCR antenna to protect it. Due to excellent physical and chemical properties, polyurethane rubber (PUR) is often used as TCR antenna baffles (hereinafter referred to as antenna baffle). Once the baffle fails, the TCR antenna will be exposed to a dangerous environment, which will bring danger to not only the normal operation of the TCR device, but also the safe operation of the train. In addition, if the antenna baffle cracks and falls on the track, it will bring derailment risk to high-speed trains. Therefore, the failure of the antenna baffle deserves attention.

Due to its high hardness, good strength, high elasticity, wear resistance, tear resistance, and aging resistance [1,2], PUR is widely used [3,4,5,6]. However, it turns out that like other polymer materials [7], PUR also has its drawbacks. Under some specific circumstances, these drawbacks are magnified. Ren et al. [8] studied the causes of inclusion defects on the surface of strip steel caused by PU rolls. In order to clarify the source of inclusions, morphology, and chemical composition of inclusions, wear debris and adhesion substances on PU rollers were analyzed in detail. Finally, it was found that the wear of the PU roller is the root cause leading to the inclusions. Junik et al. [9] researched the impact of the hardness on the selected mechanical properties of rigid PU elastomers commonly used in suspension systems. The results show that the hardness plays an important role in the fracture sensitivity of PUR material, and in a certain hardness range, PUR with higher hardness has higher toughness, wear resistance, and fatigue resistance. Wen et al. [10] found that electrostatic cumulative discharges on the surfaces of PU elastomers could lead to accidents and disasters, such as equipment failures, fires, and explosions, in industries. In order to solve this problem, they adopted the method of embedding copper foam in PU to form a copper foam-based PU composite, which greatly improved the electrostatic protection performance and wear resistance of PU. Cristiano et al. [11] developed a new methodology to study the failure of elastomers in a confined geometry and applied this new methodology to model end-linked PU elastomers. In situ experiments show that the failure of elastomers is due to the growth of a single cavity nucleated in the region of maximum hydrostatic stress. Comparison between different elastomers shows that the material containing both entanglements and crosslinks is more resistant to cavitation relative to its elastic modulus. In addition to the factors mentioned above, PUR is also affected by fatigue cracking [12,13], thermal oxidation aging [14], physical swelling [15,16], and so on. These factors have also been found in the failure of other rubber materials [17,18,19]. These studies mentioned above only focus on a specific property of PUR, and only propose corresponding solutions from a certain aspect. However, most PURs fail in complex environments, and the failure causes are often multiple rather than single. Therefore, studying the failure of PUR in specific working conditions can not only solve the actual failure problem, but also provide a reference for similar failure cases in the future.

In this paper, the material and performance of the failed baffle are analyzed by various testing methods, and its fracture surface is carefully observed by macro- and micro-observation. In addition, the root cause of the failure was verified again by experiment. Finally, it was found that the antenna baffle was exposed to the 602 cleaning agent, which caused environmental cracking stress. This is the root cause of the failed baffle. Furthermore, it was found that there were some bubbles inside the baffle, which led to the decline of its mechanical properties and made it more prone to capillary action. This is an important cause of the failed baffle. According to the above reasons, reasonable solutions are proposed. After these solutions are applied to the actual protection of the baffle, the number of failed antenna baffles is greatly reduced. This study can not only solve the failure problem of TCR antenna baffles for high-speed trains, but also help to update the understanding of the failure mechanism of PUR.

## 2. Background

**Figure 1:**
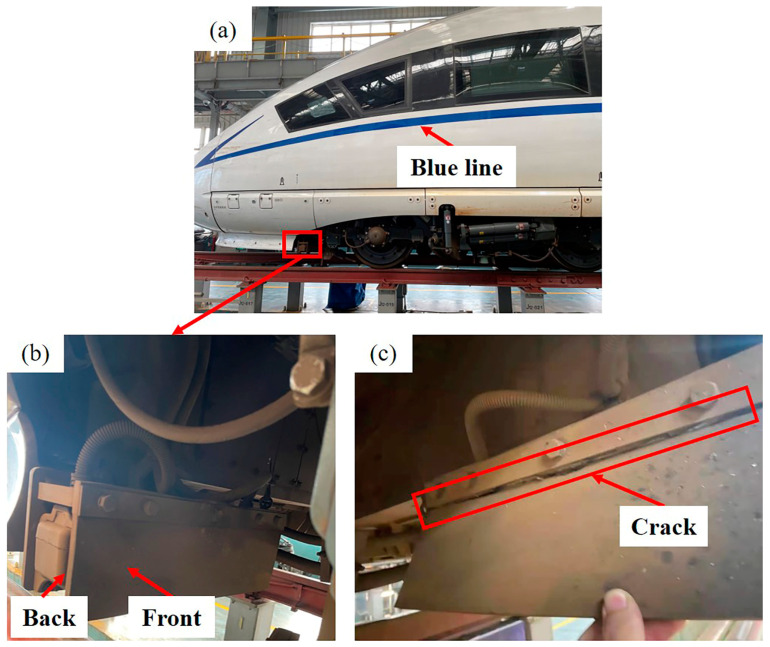
Specific location and appearance of the antenna baffle: (**a**) specific location of the baffle and the blue line; (**b**) front and back sides of the antenna baffle; (**c**) crack on the baffle.

**Figure 2:**
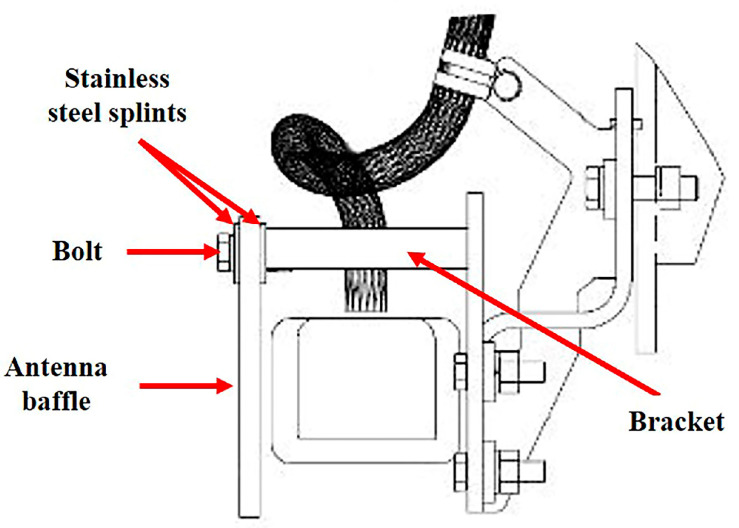
Installation diagram of the antenna baffle.

**Figure 3:**
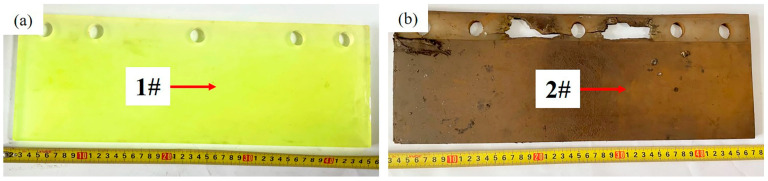
Appearance of the antenna baffles: (**a**) appearance of the new baffle; (**b**) appearance of the failed baffle.

The antenna baffle is used on the front bogie of the high-speed train, and the specific location is shown in Figure 1a. In Figure 1a, it can also be seen that there is an obvious blue line on the train body, which divides the body into upper and lower parts. Figure 1b shows the appearance of the antenna baffle. For the convenience of description, the side facing the antenna is defined as the back side, and the other side is defined as the front side. Figure 1c shows the failure of the baffle, and it can be seen that there are obvious cracks in the fixed part of the baffle. Figure 2 is an installation diagram of the antenna baffle. It shows that the antenna baffle is fixed on the bracket by two stainless steel splints and five bolts with tightening torque of 10 Nm. It is worth mentioning that the antenna baffle only exists on one side of the TCR antenna. Figure 3 shows the appearance of the light cyan new baffle and the black-brown failed baffle, which are named Sample 1# and Sample 2# in turn for the convenience of subsequent description. During the maintenance, there were two cases of antenna baffle cracking. In order to eliminate this hidden danger, the company of the high-speed train immediately carried out a special census involving 282 trains and found 21 cases of similar problems. This shows that the abnormal cracking of the antenna baffle is universal and needs to be solved urgently. In addition, the baffle was designed to have a service life of 4.8 million kilometers, but it had actually only operated 0.67 million kilometers before serious cracking occurred. Obviously, this is a premature, abnormal failure, which will bring hidden dangers to the safe operation of the train. Therefore, it is very necessary to study the cause of the premature failure.

## 3. Investigations and Discussions

### 3.1. Characterization Analysis

#### 3.1.1. Macroscopic Observation

**Figure 4:**
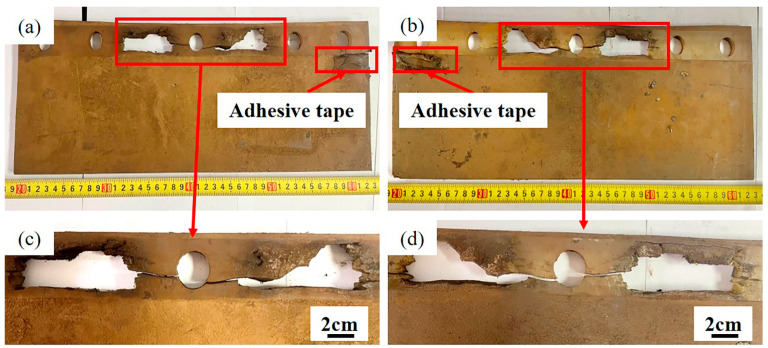
Appearance of Sample 2#: (**a**) overall appearance of the front side; (**b**) overall appearance of the back side; (**c**) cracks on the front side after partial enlargement; (**d**) cracks on the back side after partial enlargement.

Figure 4a shows the front appearance of Sample 2#. There is a lot of dust stuck to the surface of the baffle, and there is a raised strip of adhesive on the right side of the baffle, which should have been reinforced with adhesive tape after the crack was found. After partial enlargement of the front side, the cracking failure appearance is more obvious, as illustrated in Figure 4c. Figure 4b reveals the back appearance of Sample 2#. There is also adhesion on the left side. After partial enlargement of the back side, the cracking failure appearance is also obvious, as shown in Figure 4d.

#### 3.1.2. Three-Dimensional Stereomicroscope (3D-SM) Observation

**Figure 5:**
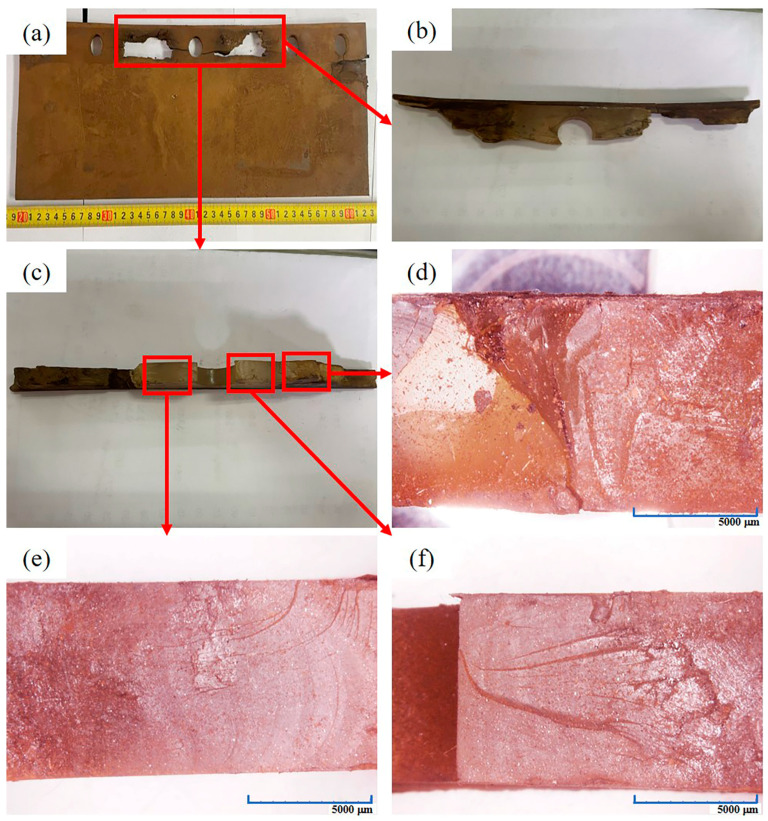
Cross-section appearance of Sample 2#: (**a**) overall appearance of Sample 2#; (**b**) sample obtained after sampling; (**c**) samples placed vertically; (**d**) appearance of the first section; (**e**) appearance of the second section; (**f**) appearance of the third section.

Figure 5a reveals the front overall appearance of Sample 2#. For the convenience of observation, the sample in the red box is cut and sampled. The front appearance of the sample after sampling is shown in Figure 5b. After placing the sample vertically (Figure 5c), the cross-section observed under 3D-SM is shown in Figure 5d–f. The three sections are greasy and bright with a lot of dust, and no obvious crack initiation point is observed. In addition, when the cross-section of the sample is touched by hand, it is obviously sticky and has low hardness (the fingernail can easily pierce into the sample), which indicates that the material has obvious performance degradation.

#### 3.1.3. Scanning Electron Microscope (SEM) Observation

In order to facilitate the observation of its morphology under SEM, Sample 2# was sonicated in deionized water for 2 h, and then dried with a blower in cold air before observation.

**Figure 6:**
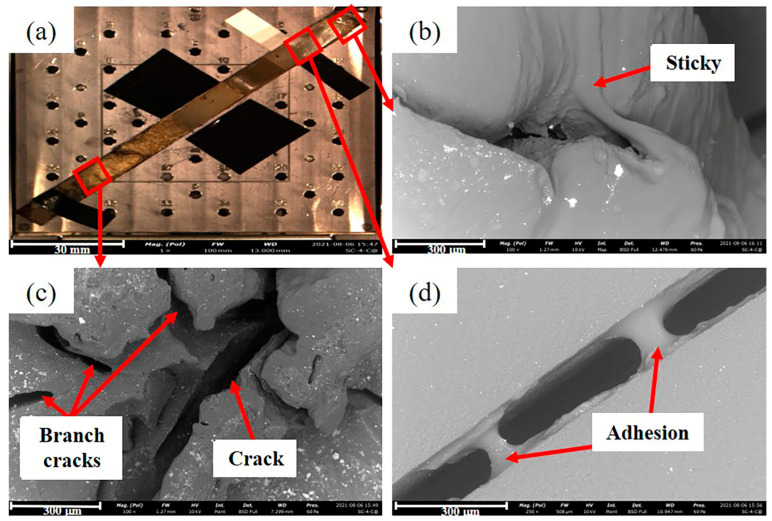
Fractograph of Sample 2#: (**a**) overall macro morphology of the section; (**b**) sticky; (**c**) main crack and branch cracks; (**d**) adhesion of the crack.

Figure 6a is the fractograph of Sample 2#. Figure 6b–d shows that the fracture of Sample 2# is characterized by sticky, cracking, and adhesion of cracks. Figure 6c shows that the main crack has many branch cracks, which is a typical environmental stress cracking (ESC) morphology. This indicates that Sample 2# was seriously affected by the external environment, resulting in a significant decrease in its performance.

### 3.2. Material Analysis

#### 3.2.1. Attenuated Total Internal Reflectance Fourier Transform Infrared Spectroscopy (ATR-FTIR) Analysis of the Antenna Baffle

**Figure 7:**
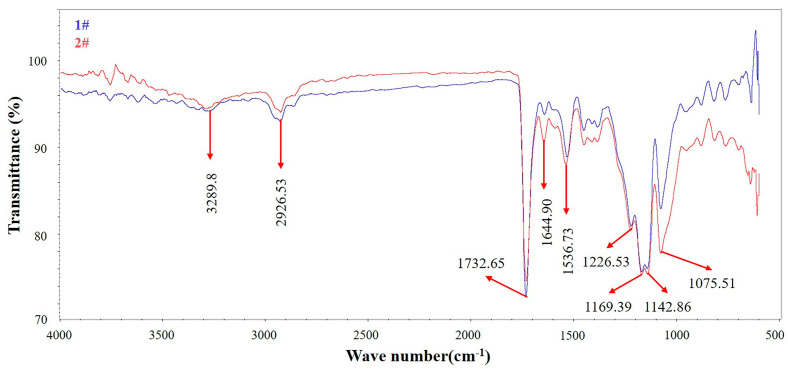
Infrared spectra of Samples 1# and 2#.

In order to determine whether new substances were produced before and after the failure, Samples 1# and 2# were analyzed by ATR-FTIR, and the analysis results are shown in Figure 7. Comparing the test results of the two samples, it can be found that the characteristic peaks of Sample 2# completely coincide with those of Sample 1#, which indicates that no chemical changes have taken place in Sample 2#. In addition, Figure 7 also shows that there are nine obvious characteristic peaks in both samples. The meaning of each characteristic peak is shown in Table 1 [20]. The existence of these characteristic peaks proves that the TCR antenna baffle is a synthetic polyurethane rubber [21,22].

#### 3.2.2. Nuclear Magnetic Resonance (NMR) Analysis of the Antenna Baffle

The NMR test results of the antenna baffle (Table 2) show that compared with the new Sample 1#, the crosslinking density of the failed Sample 2# is greatly reduced and the dispersion coefficient of the failed Sample 2# is higher. This indicates that part of the crosslinking bonds in Sample 2# is broken, and the breakage of the crosslinking bonds is not uniform. Since the FTIR test results show that the antenna baffle has no obvious chemical changes, it can be reasonably speculated that the antenna baffle may be affected by environmental stress cracking (ESC), resulting in the breakage of its crosslinking bonds.

#### 3.2.3. Thermogravimetric Analysis (TGA) of the Antenna Baffle

**Figure 8:**
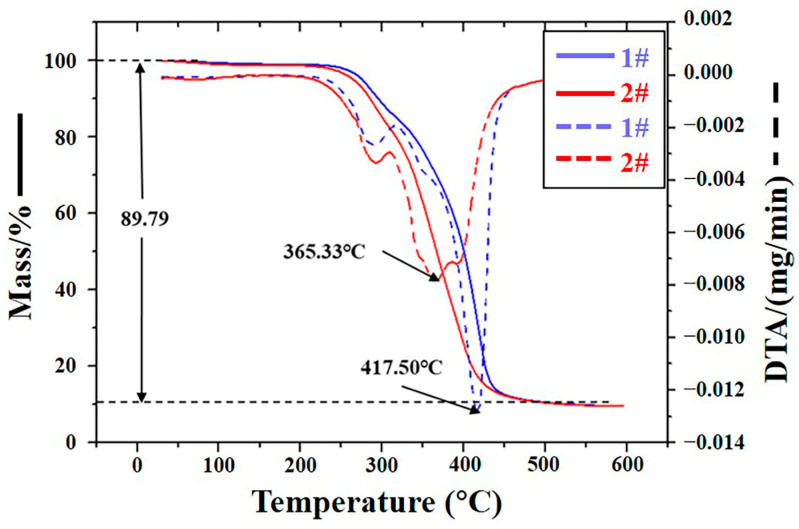
TGA curves of Samples 1# and 2#.

Figure 8 shows the thermogravimetric curve of Samples 1# and 2#. It shows that the final mass loss of Samples 1# and 2# is 89.79% (solid line in Figure 8), which indicates that there is no significant difference in mass loss between these two samples. However, by observing their mass loss rate curves (dotted line in Figure 8), it can be found that the temperature when the mass loss rate of Sample 1# reaches its maximum is 417.50 °C, and Sample 2# reaches its maximum is 365.33 °C. The difference is 52°C. That is to say, compared with Sample 1#, the temperature of Sample 2# when the mass loss rate reaches the maximum is earlier, which indicates that the heat resistance of Sample 2# is significantly reduced. Combined with the fact that the crosslinking density of Sample 2# is significantly lower than that of Sample 1#, it is believed that the ESC occurred after Sample 2# was eroded by solvent, which resulted in partial breakage of its crosslinking bonds, enhanced molecular fluidity, and decreased heat resistance.

#### 3.2.4. Differential Scanning Calorimetry (DSC) Analysis of the Antenna Baffle

**Figure 9:**
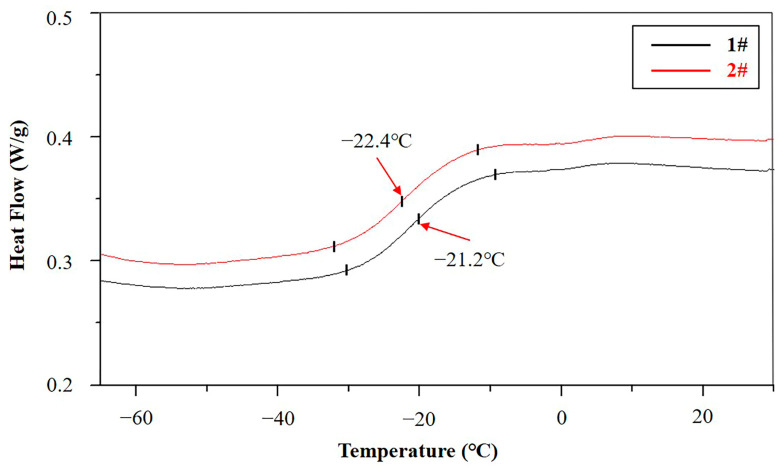
DSC curves of Samples 1# and 2#.

In order to understand the change of the glass transition temperature (Tg) before and after the failure of the antenna baffle, a DSC test was carried out on the two samples respectively, and the test results are shown in Figure 9. The Tg of Samples 1# and 2# is −21.2 °C and −22.4 °C respectively, which indicates that the Tg of Sample 1# is higher than that of Sample 2#. Combined with the NMR and TGA test results, it can be reasonably speculated that Sample 2# was affected by ESC, resulting in the breakage of its crosslinking bonds and the easier movement of the molecular chain, thus leading to the decrease in its Tg.

#### 3.2.5. Hardness Analysis of the Antenna Baffle

The Shore A hardness tester was used to test the surface hardness of Samples 1# and 2#, and the test results are shown in Table 3.

Table 3 indicates that the hardness of Sample 2# is significantly lower than that of Sample 1#, indicating that the surface of Sample 2# is obviously softened. Combined with the test results of NMR, TGA, and DSC, it can be determined that Sample 2# is affected by ESC, resulting in a great decrease in its surface hardness.

#### 3.2.6. Gas Chromatography–Mass Spectrometry (GC-MS) Analysis of the Cleaning Agents

The high-speed train is cleaned and maintained regularly, and four cleaning agents are used during cleaning. Three of them, which are named alkaline, neutral, and acidic cleaning agents, are used to clean the train body. Another cleaning agent named 602 is specially designed to clean the metal fixing parts of the bogie. It is worth mentioning that these cleaning agents are likely to contact the antenna baffle. To analyze the composition of these cleaning agents, GC-MS tests were carried out.

**Figure 10:**
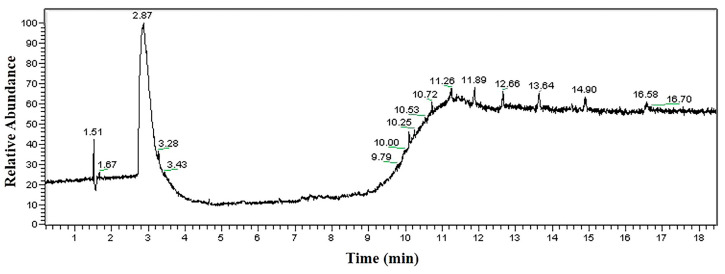
GC-MS analysis result of alkaline cleaning agent.

The analysis results of the alkaline cleaning agent are shown in Figure 10. It indicates that no matching organic compound is found.

**Figure 11:**
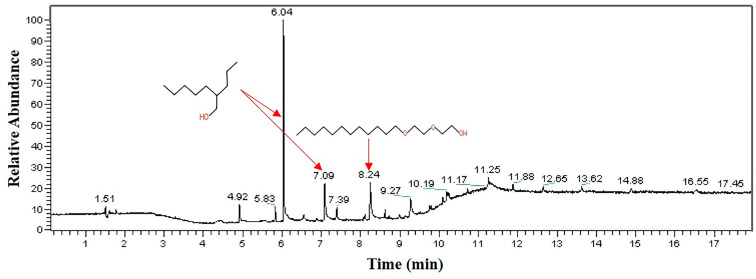
GC-MS analysis result of neutral cleaning agent.

The analysis results of the neutral cleaning agent are shown in Figure 11. There are three obvious peaks in the spectrum, and two substances are identified. The organic compounds in the neutral cleaning agent mainly contain hydroxyl and ether groups, and the substances containing the ether group account for less.

**Figure 12:**
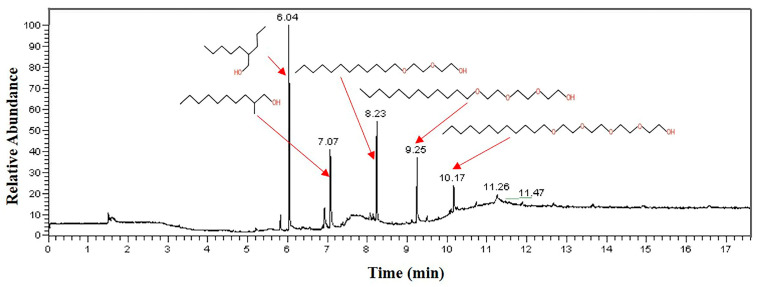
GC-MS analysis result of acidic cleaning agent.

The analysis results of the acidic cleaning agent are shown in Figure 12. The results indicate that the organic compounds in the acid cleaning agent also mainly contain hydroxyl and ether groups, and the substances containing the ether group account for less.

**Figure 13:**
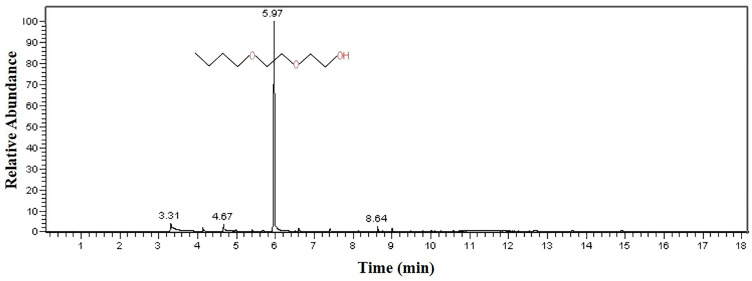
GC-MS analysis result of 602 cleaning agent.

The analysis results of the 602 cleaning agent are shown in Figure 13. The results prove that the main component of the 602 cleaning agent is 2-(2-n-butoxyethoxy) ethanol, accounting for 97.8% of the total content.

### 3.3. Soaking Experiment

In order to better understand the cleaning and maintenance of the high-speed train, a field investigation was carried out. The field investigation shows that the high-speed train must be regularly cleaned and maintained, and four cleaning agents are used: acidic, neutral, alkaline, and 602 cleaning agents. The specific usage of the four cleaning agents is shown in Table 4. Since the acidic, neutral, and alkaline cleaning agents are sprayed on a large area during the cleaning process and the 602 cleaning agent is directly sprayed on the bogie with a high-pressure spray bottle, it is reasonable to speculate that these four cleaning agents would come into contact with the failed baffle.

**Figure 14:**
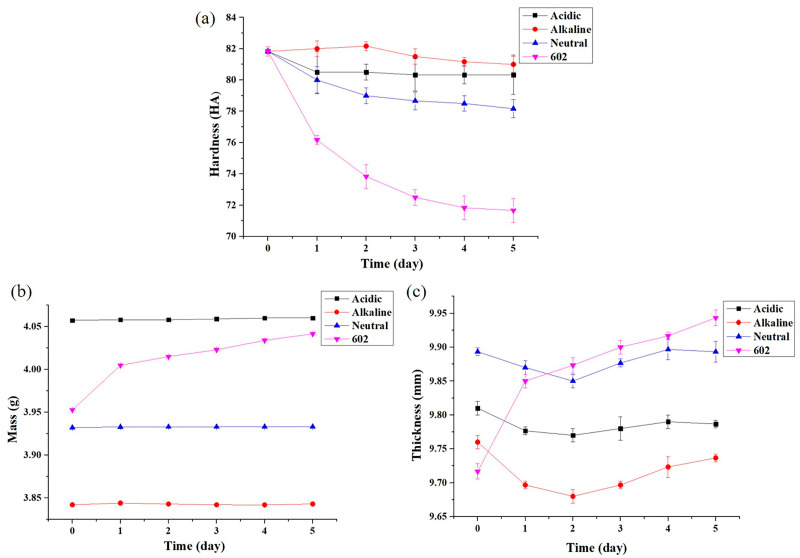
Change in hardness, mass, and thickness after soaking: (**a**) hardness; (**b**) mass; (**c**) thickness.

**Figure 15:**
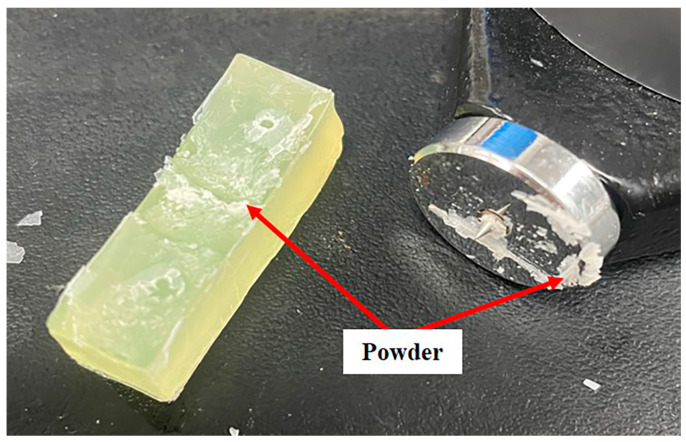
Surface changes after soaking in 602 cleaning agent.

To prove the conjecture that the failed baffle was severely corroded by the solvent, Sample 1# was cut into small pieces and soaked in different cleaning agents. The soaking results are shown in Figure 14 and Figure 15. Figure 14 shows that after soaking in alkaline, neutral, and acidic cleaning agents, the thickness, mass, and hardness of the samples changed little (within 3%). Compared with the other three cleaning agents, the thickness and mass of the sample increased after soaking in the 602 cleaning agent, but the increase ratio is also small (within 3%). What is more, the hardness of the sample decreased significantly (about 12% after soaking for 5 days). Figure 15 shows that after soaking in the 602 cleaning agent for 6 days, the surface of the sample becomes powdery. This surface change is not observed after soaking in other solvents. The experimental results indicate that the 602 cleaning agent is the key factor in the failure of the baffle.

In addition, according to the GC-MS test results, no organic matter is detected in the alkaline cleaning agent, but organic matter containing the ether group is detected in both the acidic and neutral cleaning agents. As the content of the organic matter containing the ether group in acidic and neutral cleaning agents is relatively small and has been diluted during use, the performance of the sample in acidic and neutral cleaning agents does not change significantly. The 602 cleaning agent contains 97.8% 2-(2-n-butoxyethoxy) ethanol and is used undiluted. This indicates that there is a higher concentration of organic matter containing the ether group in the 602 cleaning agent than in other cleaning agents. This is the reason why the performance of the sample in the 602 cleaning agent is seriously degraded.

## 4. Comprehensive Analysis

### 4.1. Environmental Stress Cracking

The performance test of the failed antenna baffle was carried out. The results of the FTIR test show that no obvious chemical changes occurred in the failed baffle, the results of the TGA test show that the heat resistance of the failed baffle decreases significantly, the results of the DSC test show that the Tg of the failed baffle decreases, and the results of the hardness test show that the surface hardness of the failed baffle decreases seriously. All these phenomena indicate that the failed baffle is seriously eroded by solvent. The soaking experiment results show that the hardness of the baffle decreases obviously after the baffle is exposed to the 602 cleaning agent, which further verifies that the failed baffle had the phenomenon of ESC.

ESC refers to the phenomenon in which polymers (especially glassy thermoplastics) are degraded by chemical agents in the presence of stress, resulting in damage to the polymer components. This is a solvent-induced failure as well as a material cracking that occurs in synergy between chemical agents and mechanical stress. Crystalline polymers and even lightly crosslinked polymers exhibit similar failures to glassy polymers, but generally require higher externally imposed stresses [24,25].

The reasons for ESC of the failed baffles can be explained by the following microscopic mechanism.

**Figure 16:**
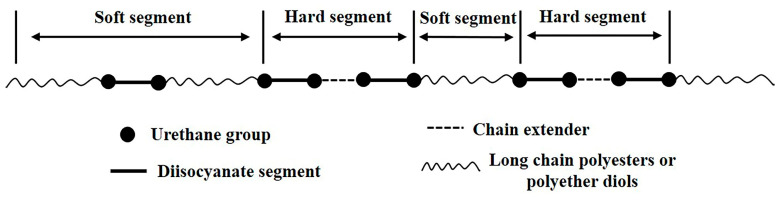
Molecular structure of PUR.

**Figure 17:**
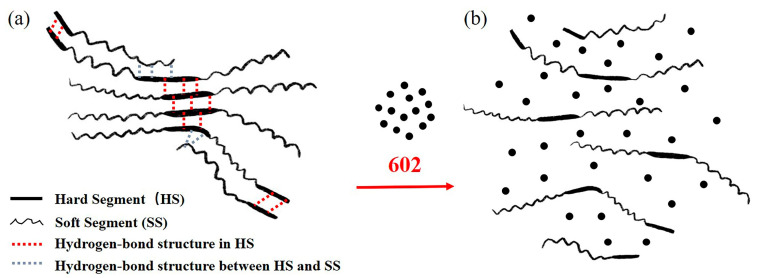
Changes in the internal structure of PUR before and after soaking: (**a**) internal structure of PUR before soaking; (**b**) internal structure of PUR after soaking.

The molecular chain of PUR contains an isocyanate group (-NCO) and a urethane group (-NHCOO-), which are obtained by polymerization of isocyanate, polyols, and chain extenders. The chain extenders include small-molecule diols, diamines, and so on. The PUR molecular chain is composed of soft and hard segments. In PUR, polyester or polyether constitutes the soft segment, while isocyanate and the crosslinking agent constitute the hard segment, as shown in Figure 16 [26]. These two segments are thermodynamically incompatible. When the hard segments are close to each other, their electron orbitals easily overlap, resulting in the formation of hydrogen bonds between the hard segments. The hard–soft hydrogen bonds are easily formed between the secondary amino group and the soft segment. [27] These intermolecular hydrogen bonds make the hard segment embedded in the soft satin, which restricts the movement of the molecular chain of the soft segment, so that the Tg of the soft satin in PUR increases with the degree of hydrogen bonding, as illustrated in Figure 17a [28]. Because of its strong polarity, the hard-segment phase in PU forms a crystalline region through the interaction of intermolecular or intramolecular hydrogen bonds, which can play a role in physical crosslinking [29,30]. Due to the combined action of the hard segment as the physical crosslinking point and soft satin with high flexibility, PUR has the advantages of impact resistance, wear resistance, high elasticity, and high elongation [31,32]. Figure 17b shows that after soaking in the 602 cleaning agent, the PU rubber is swelled, and the molecular spacing increases, resulting in the rupture of hydrogen bonds. The rupture of the hydrogen bond results in the absence of physical crosslinking between hard segments, which leads to lower heat resistance, lower Tg, lower surface hardness, and the powdery surface of the PUR.

The soaking experiment shows that the hardness of the antenna baffle decreases obviously after contacting the 602 cleaning agent. The microscopic mechanism reveals the mechanism of the decrease in the crosslinking density of PUR after soaking, which well explains the phenomenon of the surface hardness decrease and powdery surface of the baffle after soaking. More importantly, the soaking experiment and microscopic mechanism can perfectly explain the results of the Tg decrease, heat resistance decrease, crosslinking density decrease, hardness decrease, and powdery surface. This series of perfectly corroborating evidence indicated that the failed antenna baffle was exposed to the 602 cleaning agent. In addition, the high-speed train would be subjected to cyclic loads during running (as mentioned in Section 4.3 below). When solvent erosion and cyclic load are applied to the antenna baffle at the same time, ESC will occur, leading to a sharp decline in its mechanical properties. This will cause the antenna baffle to crack and fail in a short period of time. Therefore, ESC is the root cause of the premature failure of the antenna baffle.

### 4.2. Manufacturing Process

**Figure 18:**
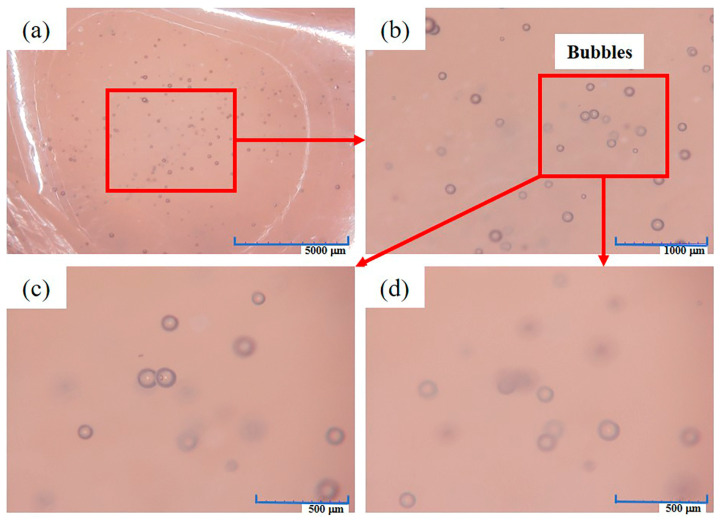
Surface appearance of Sample1# under 3D-SM: (**a**) overall appearance; (**b**) magnification; (**c**) first focal-length adjustment; (**d**) second focal-length adjustment.

The new antenna baffle (Sample 1#) was observed under 3D-SM (Figure 18). Figure 18a shows that obvious bubbles can be observed on the surface of Sample 1#. The bubbles are large (visible to the naked eye) and abundant. In addition, at 160× magnification, by adjusting the focal length of the microscope to observe the same position, it can be found that different clear bubbles can be observed at different focal lengths (Figure 18c,d). This indicates that the bubbles are not at the same height, but distributed in different layers along the thickness direction.

The existence of bubbles will reduce the mechanical properties of the antenna baffle (it is easy to produce stress concentration at the bubbles), which is not conducive to its normal use. In addition, the presence of bubbles in the antenna baffle also makes it easier for the solvent to enter, and the solvent is more likely to erode the antenna baffle due to capillarity. Therefore, some bubbles left in the antenna baffle are an important cause of the premature failure of the antenna baffle.

### 4.3. Stress

During the normal operation of the train, because the antenna baffle is fixed at one end and free at the other, it is subjected to four different forces, which are inertia force, wind resistance, gravity, and vibration force caused by an uneven roadbed [33,34]. Since PUR has the characteristics of high hardness, good strength, high elasticity, and high wear and tear resistance, these four forces will not affect the use of the baffle under normal circumstances. However, when the baffle is eroded by the solvent, the physical properties of the baffle decrease significantly, and these four forces will provide the necessary external stresses for the ESC of the baffle. It is the combination of solvent erosion and the four forces that lead to ESC. Therefore, these four forces are another important cause of the premature cracking of the antenna baffle.

## 5. Conclusions and Recommendations

On the basis of the systematic failure analysis, the main conclusions could be drawn as follows:

(1) ATR-FTIR test results show that the material of the baffle is PU, and there is no obvious chemical change before and after the failure.
(2) NMR, TGA, DSC, and hardness test results show that compared with the new baffle, the crosslinking density, heat resistance, Tg, and hardness of the failed baffle are significantly reduced, indicating that the failed baffle was swelled by solvents. After soaking in the 602 cleaning agent for a period of time, it was found that the surface hardness of the new baffle decreased significantly. Therefore, during the cleaning and maintenance of the bogie, a small amount of the 602 cleaning agent was sprayed on the baffle, causing ESC. This is the root cause of premature cracking of the antenna baffle.
(3) There are some bubbles inside the antenna baffle when it is formed, which results in the decline of some of its mechanical properties. The presence of bubbles also causes capillary action, making it easier for the cleaning agent to penetrate the baffle. This is an important cause of premature cracking of the antenna baffle.
(4) The antenna baffle withstands the forces caused by inertia, gravity, vibration force caused by uneven roadbed, and wind resistance. These four forces provide the necessary tensile and bending stresses for the cracking of the baffle. This is another important cause of premature cracking of the antenna baffle.

Based on the above conclusions, the following recommendations are put forward.

(1) When cleaning the bogie, it is necessary to cover the antenna baffle with a plastic film to prevent the 602 cleaning agent from being sprayed on the baffle. This can effectively avoid the swelling phenomenon.
(2) The supplier of antenna baffle products should improve the formulation process to ensure that the bubbles remaining in the baffle during the forming process are controlled within the acceptable quality range.
(3) The antenna baffle should avoid scratches, bumps, deformations, and other surface defects during transportation and storage. During installation, the surface state of the baffle should be carefully observed to avoid the use of defective baffles.

Finally, it is worth noting that the number of failed baffles was greatly reduced after the company adopted our solution, which proves that our recommendations are correct and effective.

## Figures and Tables

**Table 1:** Meanings of each characteristic peak [20,21,22].

Serial Number	Peak	Meaning of the Peak
1	3289 cm^−1^	Stretching vibration of N-H
2	2926 cm^−1^	Antisymmetric stretching vibration of -CH_2_ [23]
3	1732 cm^−1^	Stretching vibration of ester carbonyl C=O
4	1644 cm^−1^	Bending vibration peak of C=O in urea
5	1536 cm^−1^	Band of amide II
6	1226 cm^−1^	Stretching vibration of C-O
7	1169 cm^−1^	Stretching vibration of C-OH
8	1142 cm^−1^	Stretching vibration of C-OH
9	1075 cm^−1^	Stretching vibration of C-O in the ether group

**Table 2:** Crosslinking density of Samples 1# and 2# (10^−4^ mol/mL).

Sample	First	Second	Third	Average	Dispersion Coefficients/%
1#	19.221	18.901	19.058	19.060	0.840
2#	7.036	6.533	6.600	6.723	4.063

**Table 3:** Hardness of Samples 1# and 2# (HA).

Sample	First	Second	Third	Average
1#	83.5	84.0	82.5	83.3
2#	67.5	68.0	68.5	68.0

**Table 4:** Specific usage of the four cleaning agents.

Cleaning Agent	Main Ingredients	Concentration When Used	Cleaning Location	Cleaning Frequency
Alkaline	/	20%	Below the blue line (Figure 1)	Four days
Neutral	Hydroxyl organics	20%	Above the blue line (Figure 1)	Four days
Acid	Hydroxyl organics	30%	All the body of the train	Three months
602	2-(2-n-Butoxyethoxy) ethanol	100%	Metal parts of the bogie	Overhaul

## Data Availability

Not applicable.
